# Localization of the PelC and PelE effectors to the *Legionella*-containing vacuole through host-mediated prenylation and their role in intracellular proliferation

**DOI:** 10.1128/iai.00605-25

**Published:** 2026-03-24

**Authors:** Cheon Jee Shin, Christopher T. D. Price, Manal S. J. Da'as, Yousef Abu Kwaik

**Affiliations:** 1 Department of Microbiology and Immunology, University of Louisville 5170, Louisville, Kentucky, USA; 2 Center for Predictive Medicine, University of Louisville 5170, Louisville, Kentucky, USA; University of Illinois Chicago, Chicago, Illinois, USA

**Keywords:** prenylation, Prenylated Effectors of *Legionella *(Pel), intracellular, trafficking, lysosomes

## Abstract

*Legionella pneumophila* is an intracellular pathogen that replicates in macrophage and protozoan hosts within an endoplasmic reticulum (ER)-derived phagosome that evades the endosomal-lysosomal degradation pathway. The Dot/Icm Type IV Secretion System (T4SS) of *L. pneumophila* injects ~368 effector proteins into eukaryotic host cells to manipulate various cellular processes, remodeling macrophages and protozoan hosts into proliferative niches. However, most *L. pneumophila* effectors are dispensable for intracellular bacterial proliferation. Many of the effectors contain eukaryotic-like domains and motifs, such as the C-terminal “-CaaX” prenylation motif. In *L. pneumophila* strain AA100/130b, there are seven effectors harboring C-terminal -CaaX motif, designated as prenylated effectors of *Legionella* (Pels). Ectopic expression of Pels in mammalian cells results in distinct membrane localization, yet their subcellular localization and their role in the infection remain unknown. Here, we show spatial localization of the seven Pels to the cytosolic face of the LCV in a T4SS-dependent manner, and the conserved cysteine residue of the -CaaX motif is required for this spatial localization. Null mutations in *pelC* and *pelE* resulted in a significant reduction in intracellular replication in human monocyte-derived macrophages (hMDMs) and *Acanthamoeba polyphaga*, but not in *Vermamoeba vermiformis*, exhibiting host tropism. The *ΔpelC* and *ΔpelE* mutants exhibited significant failure in remodeling of the LCV by the ER and a significant increase in trafficking of their vacuoles through the endosomal-lysosomal degradation pathway within human macrophages. Our findings indicate that the PelC and PelE effectors, which hijack the highly conserved eukaryotic prenylation machinery, are required for LCV biogenesis and for intracellular replication.

## INTRODUCTION

*Legionella pneumophila* is a Gram-negative aquatic bacterium found in natural and man-made water sources (1). While protozoa feed on most bacteria as a source of nutrition, *L. pneumophila* has evolved to infect and proliferate within a wide range of protozoan species as the natural hosts (2, 3). Upon entry into amoeba hosts, *L. pneumophila* is enclosed within endoplasmic reticulum (ER)-derived vacuole that evades the endosomal-lysosomal pathway, designated as the *Legionella*-containing vacuole (LCV) (2). Proliferation of *L. pneumophila* within the natural protozoan hosts facilitates bacterial persistence and amplification of *L. pneumophila* in the environment and enhances virulence (3, 4). *L. pneumophila* released from protozoan hosts shows increased infectivity of human macrophages, high motility, and increased resistance to antibiotics, biocides, and disinfectants (5–8). Since phagocytosis and trafficking through the endosomal-lysosomal degradation pathway are highly conserved through evolution from unicellular eukaryotes to mammals, the co-evolution of *L. pneumophila* with protozoan hosts has most likely enhanced *L. pneumophila* infectivity in macrophages of the accidental human host (3). Interestingly, amoebae are thought to be the evolutionary ancestors of macrophages (9, 10).

Upon transmission to humans via aerosols generated from contaminated water sources, the bacteria infect and proliferate in alveolar macrophages (11). The biogenesis of the LCV into an ER-derived phagosome that evades phagosome-lysosome fusion within protozoa and macrophages is totally dependent on the Dot/Icm Type IV Secretion System (T4SS) of *L. pneumophila*, which functions as a biological nano-syringe to inject ~368 different effector proteins into the host cell (12–15). The long-term co-evolution and adaptation of *L. pneumophila* to various protozoan hosts resulted in accumulation of a large number of effector proteins with highly conserved eukaryotic-like domains and motifs, such as a F-box domain, U-box domain, ankyrin repeats, and prenylation motif (2, 3, 15–17).

Prenylation (farnesylation or geranylgeranylation) is one of the highly conserved post-translational modifications of eukaryotic proteins that involves the modification of the C-termini of hydrophilic proteins by lipidation, enabling their anchoring into the lipid bilayer of cellular membranes (18). Prenylation and anchoring into a specific membrane are essential for functional activity of various prenylated eukaryotic proteins, such as Rab proteins, Ras proteins, G proteins, and protein kinases (19–22). Prenylation involves the addition of a covalently bonded 15- or 20-carbon isoprenoid moiety to the conserved cysteine residue of “-CaaX” motif at the −4 position from the C-terminus. After prenylation of the cysteine residue, the -aaX tripeptide is cleaved by the Ras-converting enzyme-1 (RCE-1) protease in the ER membrane. The farnesylated cysteine is then methylated by isoprenyl cysteine carboxyl methyltransferase (ICMT) (23). We have previously shown that all three farnesylation enzymes are acquired by the LCV in a Dot/Icm-dependent manner, and the LCV membrane is decorated with farnesylated proteins (24).

Bioinformatic genome analysis of the genome of four commonly studied *L. pneumophila* strains (Philadelphia, Paris, Lens, and Corby) revealed at least 11 effector proteins that are prenylated during ectopic expression in human cell lines; these were designated Pels or CAAX motif proteins (CMPs) (17, 25). The AA100/130b strain harbors seven prenylated effector genes encoding PelA, PelC, PelD, PelE, PelH, PelI, and PelK (17). Previous studies have shown that these proteins are localized to distinct membranes during ectopic expression (17, 25). However, subcellular localization and the role of the seven Pel effectors in infection of macrophages and various amoeba hosts are not known.

The first characterized prenylated *L. pneumophila* effector protein is Ankyrin B (AnkB), which is anchored to the LCV membrane by host-mediated farnesylation, and LCV localization of AnkB is essential for intracellular bacterial proliferation (24, 26). Another characterized prenylated effector is PelE/LegG1/MitF, which has homology to the Ras-related nuclear protein (Ran) guanine nucleotide exchange factor (GEF) known as regulator of chromosome condensation 1 (RCC1) (27, 28). Ectopically expressed Ran and its effector RanBP1 have been shown to co-localize with PelE/LegG1/MitF to the LCV membrane in a Dot/Icm-dependent manner during *L. pneumophila* infection of *Dictyostelium discoideum* (27). It has been shown that PelE/LegG1/MitF stabilizes microtubules within *D. discoideum* and the mouse macrophage cell line (RAW264.7), allowing LCVs to move along the microtubules (27). In addition, PelE/LegG1/MitF has also been shown to induce mitochondrial fragmentation in human monocyte-derived macrophages (hMDMs) during infection, and *L. pneumophila*-mediated mitochondrial fragmentation occurs in a dynamic 1-like protein (DNM1L)-dependent manner (29). This mitochondrial fragmentation leads to impaired oxidative phosphorylation, which favors intracellular replication of *L. pneumophila*, as pharmacological inhibition of DNM1L using Mdivi1 to block mitochondrial fragmentation significantly reduced intracellular replication of WT *L. pneumophila* in hMDMs (29). However, direct analysis of the effector PelE/LegG1/MitF contributing to intracellular replication was not tested.

Since the Pel effectors contain a highly conserved eukaryotic motif, it is likely that *L. pneumophila* acquired the Pel-encoding genes through interkingdom horizontal gene transfer during the long coevolution of *L. pneumophila* with the protozoan hosts (2, 3). Since the Pel effectors hijack the highly conserved prenylation machinery, it is likely that these effectors are required for infection and intracellular replication of *L. pneumophila* within macrophages and amoeba hosts. Here, we show that the -CaaX motif of the seven Pel effectors (PelA, PelC, PelD, PelE, PelH, PelI, and PelK) is essential for spatial localization of the Pel effectors to the cytosolic face of the LCV membrane within hMDMs. While most *L. pneumophila* effectors are dispensable for infection of macrophages, the PelC and PelE effectors are required for intracellular replication of *L. pneumophila* in hMDMs. However, the two effectors exhibit host tropism among amoeba hosts since they are required in *A. polyphaga* but dispensable in *V. vermiformis*. Importantly, our data show that PelC and PelE effectors play a role in ER-mediated remodeling and lysosomal evasion by the LCV.

## RESULTS

### Localization of the Pels to the cytosolic face of the LCV

Previous studies have shown that ectopically expressed Pels in HEK293 and COS-1 cells are localized to either the plasma membrane or vesicular membrane (17, 25). However, pathogenic effectors that are injected into host cells by the bacterial T3SS or T4SS exhibit distinct subcellular localization during infection compared to ectopic expression. For example, while the prenylated AnkB effector is localized to the plasma membrane during ectopic expression, it is exclusively LCV-localized during infection (24). Since the cysteine residue of the eukaryotic C-terminal -CaaX motif is prenylated and is essential for anchoring prenylated proteins to membranes, we generated *L. pneumophila* strains that express the N-terminus hemagglutinin (4HA)-tagged Pel proteins downstream of an isopropyl β-D-1-thiogalactopyranoside (IPTG)-inducible promoter (18). We also constructed 4HA-Pels in which the conserved cysteine residue was substituted with alanine (CaaX → AaaX). The proper equivalent expression of these fusion proteins was validated by Western blot analysis (Fig. S1).

**Fig 1:**
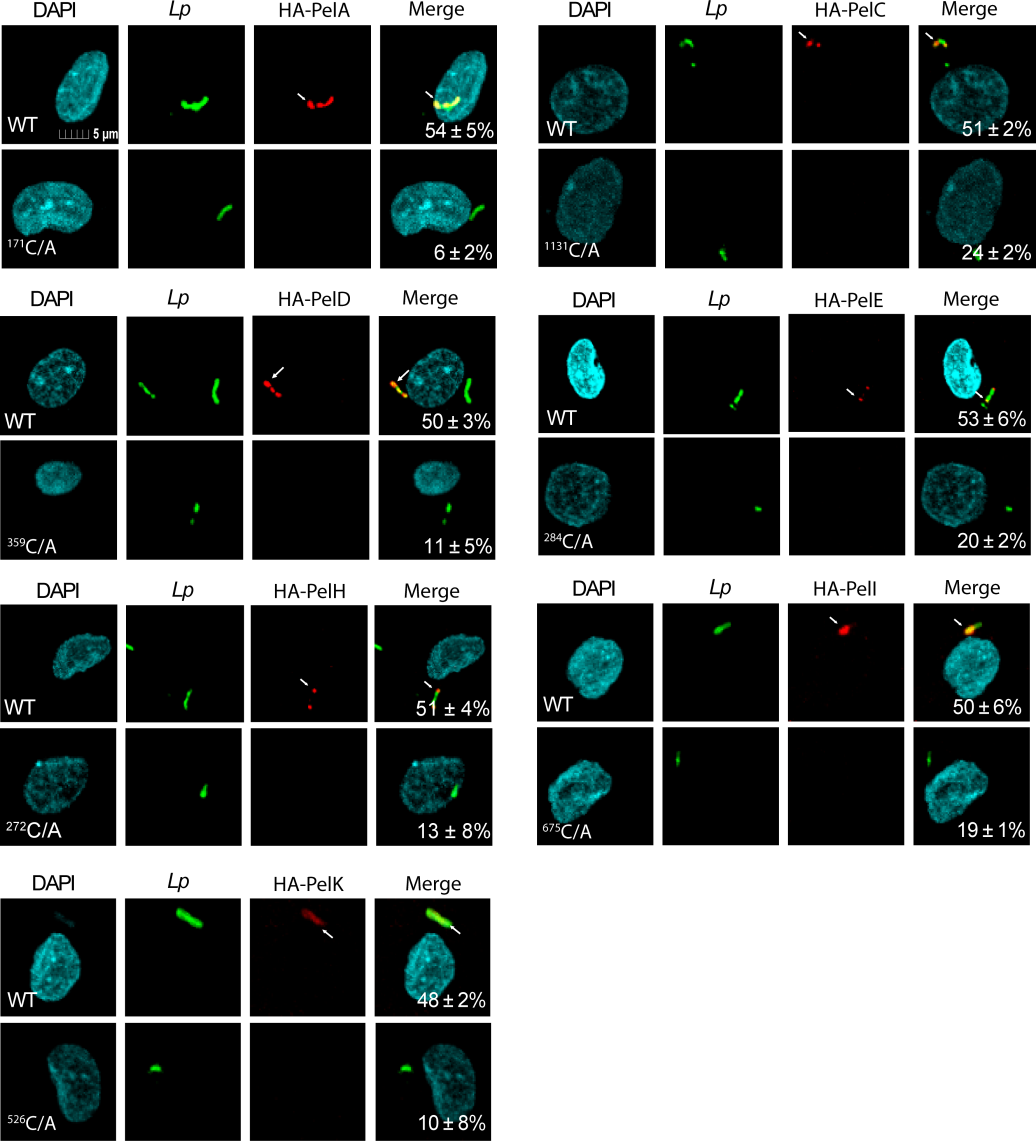
The CaaX prenylation motif of the Pels is essential for LCV localization. To determine if prenylation is required for the correct subcellular localization of the Pels during the infection of hMDMs, cysteine residues in the -CaaX motif of 4HA-tagged Pels constructs were substituted to alanine. Images are representative confocal microscopy images of 4HA-Pels (red) colocalizing with the bacteria (green) at 8 h post-infection. All analyses were performed on at least 100 infected cells from multiple coverslips. Numbers in the merged images are the mean percent colocalization of HA-tagged fusion proteins with the LCVs ± standard deviations (SD) and are representative of three independent experiments performed in triplicates. The mean percent colocalization values between WT and C/A Pels are significantly different by one-way ANOVA (*P* ≤ 0.0001).

To determine subcellular localization of the Pel effectors during infection of hMDMs and the role of the -CaaX motif in proper localization of the Pels, the plasma membranes of infected hMDMs were selectively permeabilized using 0.1% Triton X-100 as previously described (30). This approach allows loading the cytosol with antibodies while preserving the integrity of intracellular membranes, such as the LCV. The LCV was labeled using anti-*L*. *pneumophila* anti-serum, which has been employed in multiple prior studies (24, 30–34). Our data showed that all seven Pels were spatially localized to ~50% of the LCV membrane. Importantly, substitution of the conserved cysteine residue to alanine resulted in a significant reduction in localization of the Pel effectors to the cytosolic face of the LCV compared to native Pels translocated by WT bacteria (one-way ANOVA, *P* < 0.0001) (Fig. 1). To exclude the potential effect of Triton on permeabilization of the LCV membrane, methanol fixation and permeabilization were utilized as methanol permeabilizes both plasma membrane and all intracellular membranes. In methanol-treated cells, there was no significant difference in localization of the Pels between the WT Pels and the C/A mutant Pels (one-way ANOVA, *P* > 0.1) (Fig. S2).

**Fig 2:**
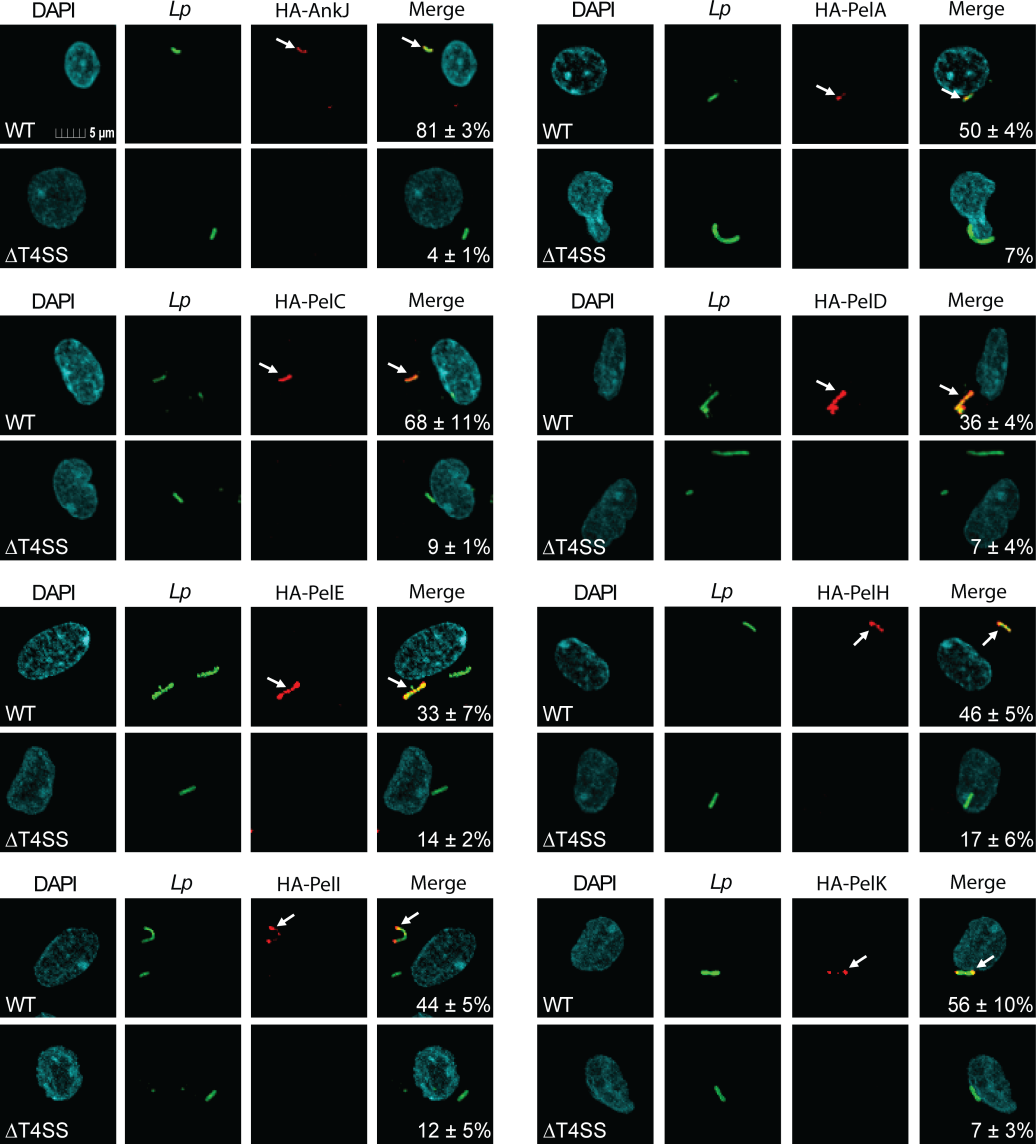
Localization of Pels to the LCV membrane during hMDM infection. To determine the subcellular localization of the Pels during the infection of hMDMs, 4HA-tagged Pels constructs were transformed into WT and *ΔT4SS L. pneumophila*. Images are representative confocal microscopy images of 4HA-Pels (red) colocalizing with the bacteria (green) at 8 h post-infection. All analyses were performed on at least 100 infected cells from multiple coverslips. Numbers in the merged images are the mean percent colocalization of HA-tagged fusion proteins with the LCVs ± standard deviations (SD) and are representative of three independent experiments performed in triplicates. The mean percent colocalization of HA-Pels with the LCVs between WT and *ΔT4SS L. pneumophila* is significantly different by one-way ANOVA (*P* ≤ 0.0001).

To determine whether the localization of the Pels was Dot/Icm-dependent, we constructed *L. pneumophila* strains that express 4HA-Pels in WT and *ΔT4SS* strains. We confirmed the proper equivalent expression of these reporter fusion proteins by Western blot analysis of total bacterial lysates (Fig. S3). The LCV-localized effector AnkJ was used as a control. By confocal microscopy, the 4HA-AnkJ reporter control of WT bacteria was localized to 81% of the LCVs and to 4% of the *ΔT4SS* mutant strain (one-way ANOVA, *P* < 0.0001) (Fig. 2). The 4HA-PelC translocated by WT bacteria was localized to 68% of the LCVs, while it was only detected in 9% of the *ΔT4SS* mutant strain (one-way ANOVA, *P* < 0.0001). The other six Pel reporters translocated by WT bacteria showed 33%–56% localization to the LCV, which was significantly less in the *ΔT4SS* mutant strains (7%–17%) (one-way ANOVA, *P* < 0.0001). Similar findings have been reported in other studies that also observed low levels of effector detection in *ΔT4SS* mutants (24, 35–37). We conclude that the Dot/Icm-dependent translocated Pel effectors are anchored to the cytosolic face of the LCV membrane, and the conserved cysteine residue of the -CaaX prenylation motif is essential for LCV localization during infection of human macrophages by *L. pneumophila* (Fig. 1 and 2).

### Role of the Pel effectors in intracellular replication

**Fig 3:**
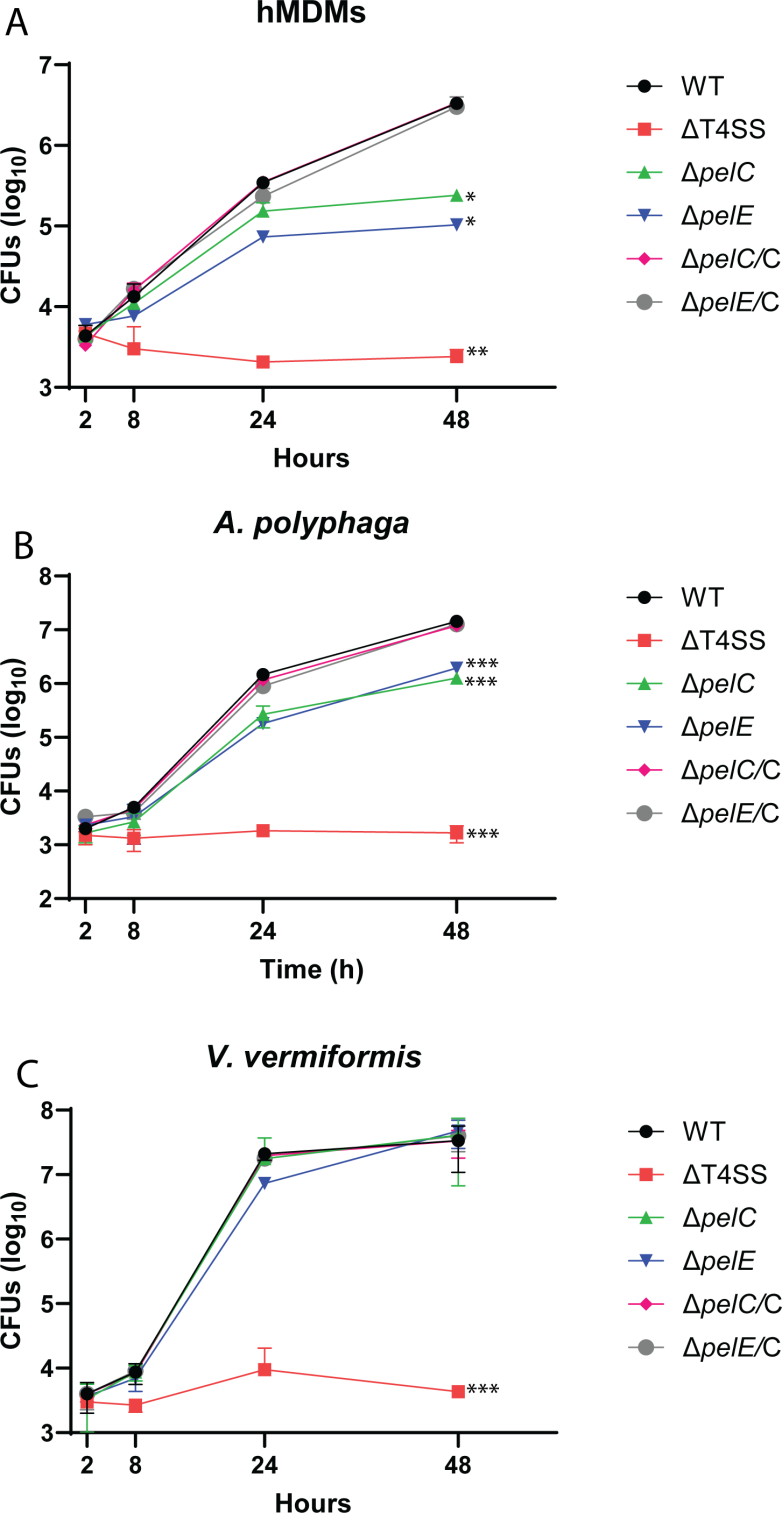
Role of PelC and PelE in intracellular replication. To determine the role of PelC and PelE in intracellular replication, (**A**) hMDMs, (**B**) *A. polyphaga*, and (**C**) *V. vermiformis* were infected. The number of CFU was determined at 2, 8, 24, and 48 h post-infection. Data points represent mean CFU ± (SD) (error bars) and are representative of three independent experiments performed in triplicates. Values that are significantly different by Student’s *t*-test are indicated as follows: * (*P* ≤ 0.05), ** (*P* ≤ 0.01), *** (*P* ≤ 0.001).

Most effectors of *L. pneumophila* are not required for intracellular proliferation in macrophages, and the role of most of them in infection of protozoan hosts is not known (2). It is most likely that the effectors that exploit highly conserved eukaryotic processes are required for intracellular replication in macrophages and amoeba. Since the Pel effectors exploit the highly conserved prenylation machinery, we determined whether the Pels were required for intracellular replication. Isogenic mutants of the Pels were generated and used to infect hMDMs, *A. polyphaga*, and *V. vermiformis*. The intracellular CFUs were enumerated after 2, 8, 24, and 48 h post-infection. There were no detectable changes in cell viability or morphology of the cells infected by any of the mutants vs the WT strain. The data showed that five of the seven null mutants (*ΔpelA*, *ΔpelD*, *ΔpelH*, *ΔpelI*, and *ΔpelK*) showed no significant growth defect during the infections of hMDMs, *A. polyphaga*, and *V. vermiformis* (Student’s *t*-test, *P* > 0.1) (Fig. S4 to S6). However, the *ΔpelC* and *ΔpelE* mutants showed more than 10-fold decrease in intracellular replication by 48 h of infection of hMDMs (Student’s *t*-test, *P* < 0.05) (Fig. 3A). Complementation of the two mutants on a plasmid (*ΔpelC/*C and *ΔpelE/*E) restored the intracellular growth defect similar to the WT strain (Student’s *t*-test, *P* > 0.1). Since the AlphaFold-predicted structures and amino acid sequence of the primary structures of PelC and PelE have no detectable similarities, it is highly unlikely that the two distinct proteins have the same biological function.

Similar to hMDMs, infection of *A. polyphaga* also showed defective phenotype for the *ΔpelC* and *ΔpelE* mutants after 24 and 48 h post-infection, and the defect was restored in the complemented strains (Student’s *t*-test, *P* < 0.0005) (Fig. 3B). Interestingly, none of the seven *Δpel* mutants exhibited a detectable defect upon infection of *V. vermiformis*, which is the most predominant non-pathogenic amoeba in natural water resources (Student’s *t*-test, *P* > 0.1) (Fig. 3C) (38). These data show that the LCV-localized PelC and PelE effectors play a role in intracellular replication of *L. pneumophila* within hMDMs and exhibit host tropism among amoeba hosts.

### Role of PelC and PelE in ER-mediated remodeling and lysosomal evasion by the LCV

**Fig 4:**
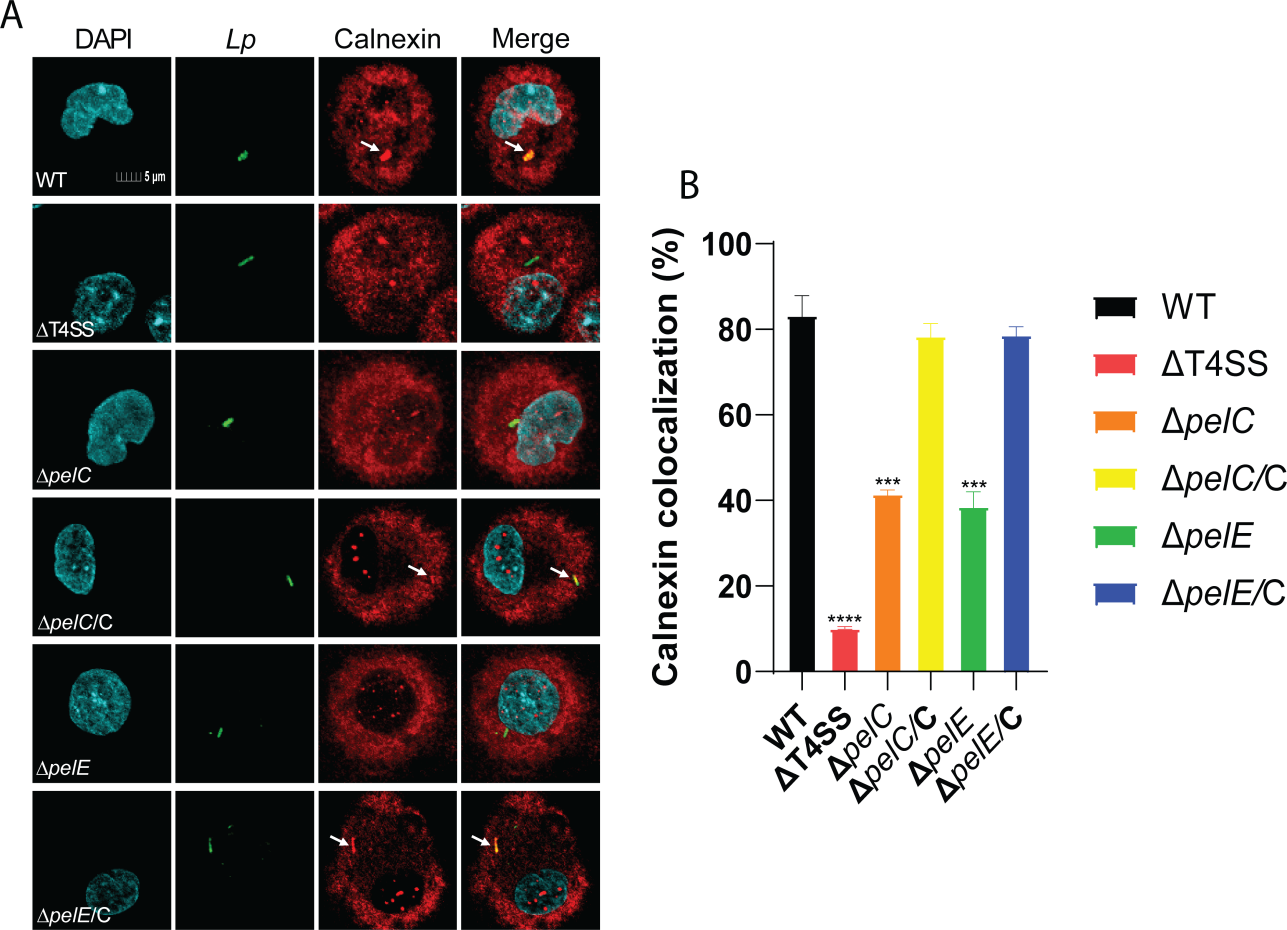
Role for PelC and PelE in remodeling the LCVs with ER proteins. To determine whether PelC and PelE are required for ER-mediated remodeling of the LCV, hMDMs were infected with WT, *ΔT4SS*, *ΔpelC*, *ΔpelE*, and the complemented *pelC*/C and *pelE*/E mutant strains. (**A**) Representative images of colocalization of the LCVs with the ER marker Calnexin. Cells are labeled with DAPI (cyan), anti-*L*. *pneumophila* (green), and anti-Calnexin (red). (**B**) Quantification of colocalization with the LCV. All analyses were performed on at least 100 infected cells from three coverslips. Data are shown as mean percent colocalization of Calnexin with the LCVs ± SD (error bars) and are representative of three independent experiments performed in triplicates. Values that are significantly different by one-way ANOVA are indicated as follows: *** (*P* ≤ 0.001), **** (*P* ≤ 0.0001).

Once phagocytosed into a host cell, the LCV intercepts ER secretory vesicles to remodel its phagosome into an ER-derived vacuole that evades the endosomal-lysosomal degradation pathway, which is essential for intracellular proliferation of *L. pneumophila* (3, 39). Since *ΔpelC* and *ΔpelE* mutants showed defective intracellular growth in hMDMs, we determined whether trafficking of the LCVs harboring the two mutants was altered. First, we determined whether the *ΔpelC* and *ΔpelE* mutants failed to create an ER-derived vacuole by assessing colocalization of the LCV with the ER resident protein Calnexin (40). Our data showed that by 2 h post-infection of hMDMs, 83% of the LCVs harboring WT bacteria colocalized with Calnexin, compared to only 10% of the LCVs harboring the translocation-defective *ΔT4SS* mutant colocalized with the ER marker (one-way ANOVA, *P* < 0.0001) (Fig. 4A and B). In contrast, 42% and 39% of the *ΔpelC* and *ΔpelE* mutant-containing vacuoles colocalized with Calnexin, which was significantly less than the WT strain (one-way ANOVA, *P* < 0.0005) (Fig. 4A and B). Similar to the WT strain-containing LCVs, ~80% of the complemented mutants (*ΔpelC*/C and *ΔpelE*/E) containing LCVs colocalized with Calnexin (one-way ANOVA, *P* > 0.1) (Fig. 4A and B).

**Fig 5:**
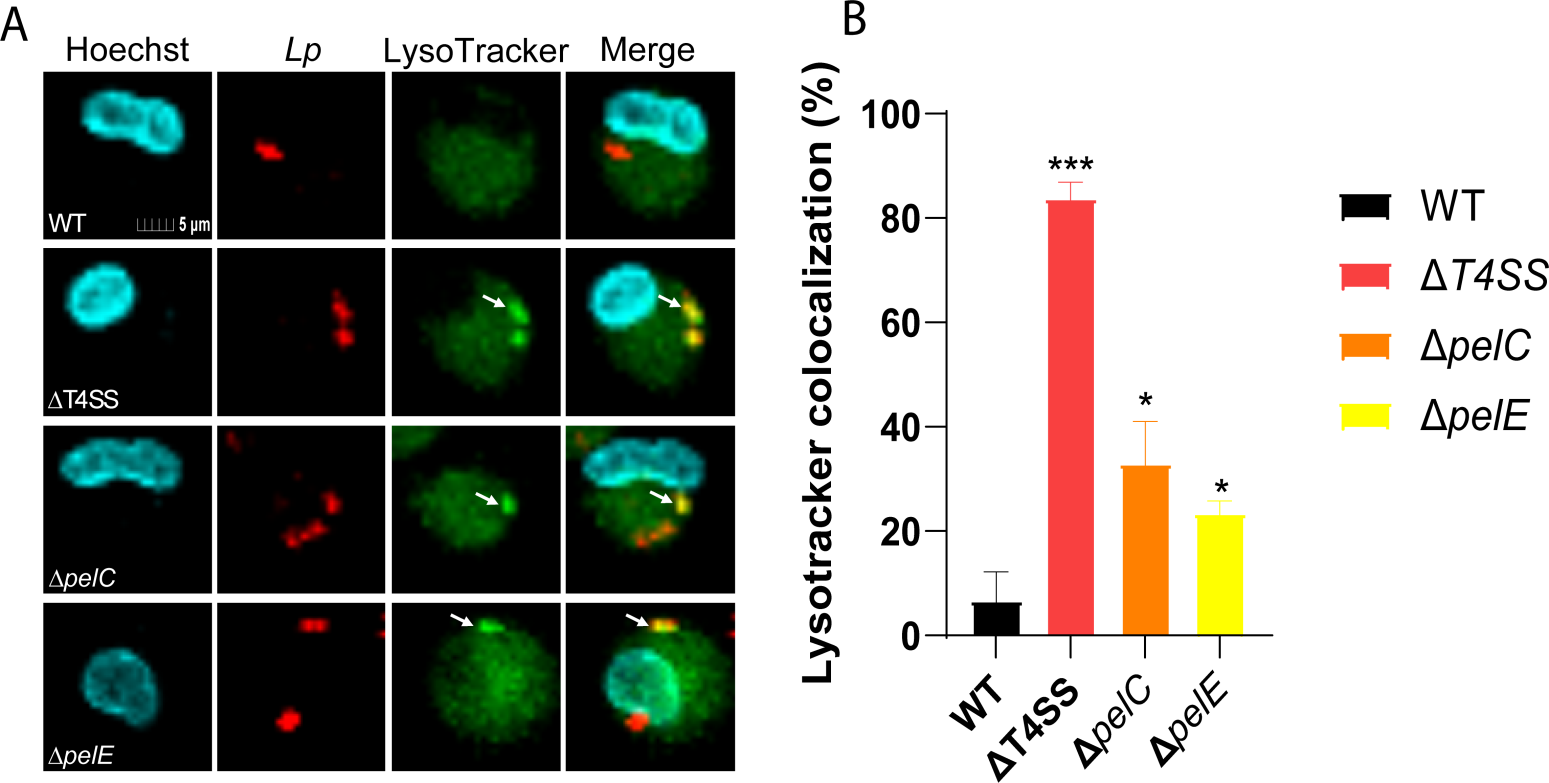
Role for PelC and PelE in evasion of acidification of the LCVs. To determine whether PelC and PelE are required for lysosomal evasion of the LCV, hMDMs were infected with mCherry expressing WT, *ΔT4SS*, *ΔpelC*, and *ΔpelE L. pneumophila* strains. LysoTracker Green DND-26 was added to the wells, and live cells were imaged by confocal microscopy. (**A**) Representative images of colocalization of the LCVs with LysoTracker. Cells are labeled with Hoechst (cyan), anti-*L*. *pneumophila* (red), and LysoTracker (green). (**B**) Quantification of colocalization with the LCV. All analyses were performed on at least 100 infected cells from multiple coverslips. Data are shown as mean percent colocalization of LysoTracker with the LCVs ± SD (error bars) and are representative of three independent experiments performed in triplicates. Values that are significantly different by one-way ANOVA are indicated as follows: * (*P* ≤ 0.05), *** (*P* ≤ 0.001).

Next, we determined whether the LCVs of the *ΔpelC* and *ΔpelE* mutants were trafficked through the endosomal-lysosomal degradation pathway. First, we utilized live-cell imaging to assess colocalization of the LCV using LysoTracker Green, which accumulates within acidified vacuoles (41). The hMDMs were infected with *L. pneumophila* strains for 30 min, and LysoTracker was added to the cells for an additional 30 min. Our data showed that at 1 h post-infection, the LCVs of the WT strain showed ~7% colocalization with the LysoTracker, while the *ΔT4SS* mutant showed 84% colocalization with LysoTracker (one-way ANOVA, *P* < 0.0004) (Fig. 5A and B). The *ΔpelC* and *ΔpelE* mutants showed 33% and 23% colocalization, respectively, which was significantly more than the WT strain (one-way ANOVA, *P* < 0.05) (Fig. 5A and B). Complementation of *pelC* and *pelE* could not be performed in this experiment as the *L*. pneumophila strains used carry an mCherry-expressing plasmid for live-cell imaging. Both the mCherry and the complementation plasmids share the same origin of replication, precluding their simultaneous use.

**Fig 6:**
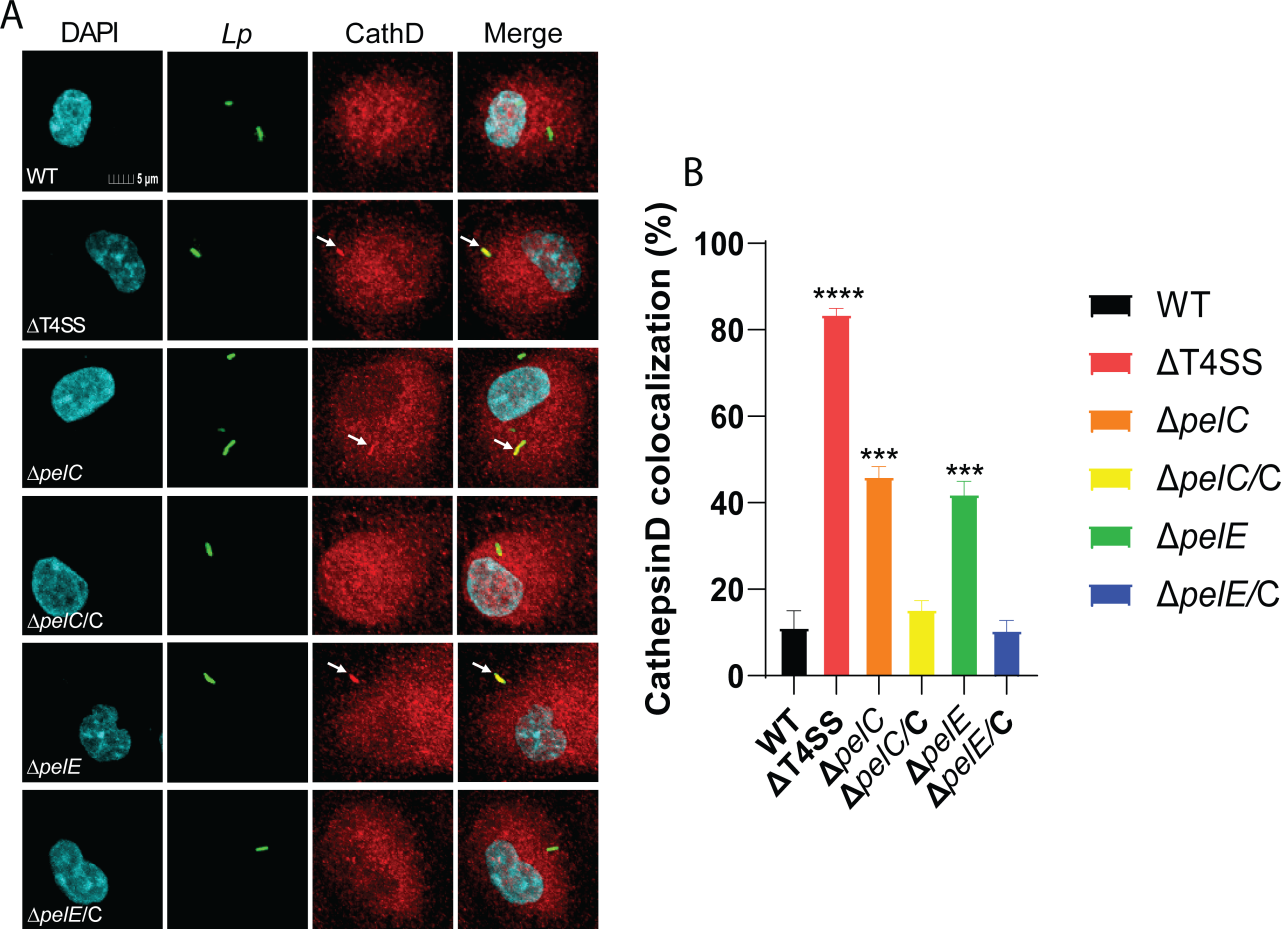
Role for PelC and PelE in evasion of lysosomal enzymes by the LCV. To determine whether PelC and PelE are required for lysosomal evasion of the LCV, hMDMs were infected with WT, *ΔT4SS*, *ΔpelC*, *ΔpelE*, and the complemented *pelC*/C and *pelE*/E strains. (**A**) Representative images of colocalization of the LCVs with the lysosomal marker Cathepsin D. Cells are labeled with DAPI (cyan), anti-*L*. *pneumophila* (green), and anti-Cathepsin D (red). (**B**) Quantification of colocalization with the LCV. All analyses were performed on at least 100 infected cells from multiple coverslips. Data are shown as mean percent colocalization of Cathepsin D with the LCVs ± SD (error bars) and are representative of three independent experiments performed in triplicates. Values that are significantly different by one-way ANOVA are indicated as follows: *** (*P* ≤ 0.001), **** (*P* ≤ 0.0001).

To evaluate lysosomal fusion of LCV harboring the two mutants, we utilized the lysosomal enzyme, Cathepsin D, as a marker for lysosomal fusion (41). Our data showed that only 11% of the LCVs harboring WT bacteria colocalized with Cathepsin D, while 84% of the *ΔT4SS* LCVs showed colocalization with Cathepsin D (one-way ANOVA, *P* < 0.0001) (Fig. 6A and B). The data showed that 46% and 42% of the LCVs harboring *ΔpelC* and *ΔpelE* mutants, respectively, colocalized with Cathepsin D, which were significantly more than the WT strain LCVs (one-way ANOVA, *P* < 0.0001) (Fig. 6A and B). The defective phenotype of the mutants was restored upon complementation of the respective WT gene, where only 15% and 11% of the vacuoles harboring the complemented *pelC* and *pelE* mutants colocalized with Cathepsin D (one-way ANOVA, *P* > 0.1) (Fig. 6A and B).

**Fig 7:**
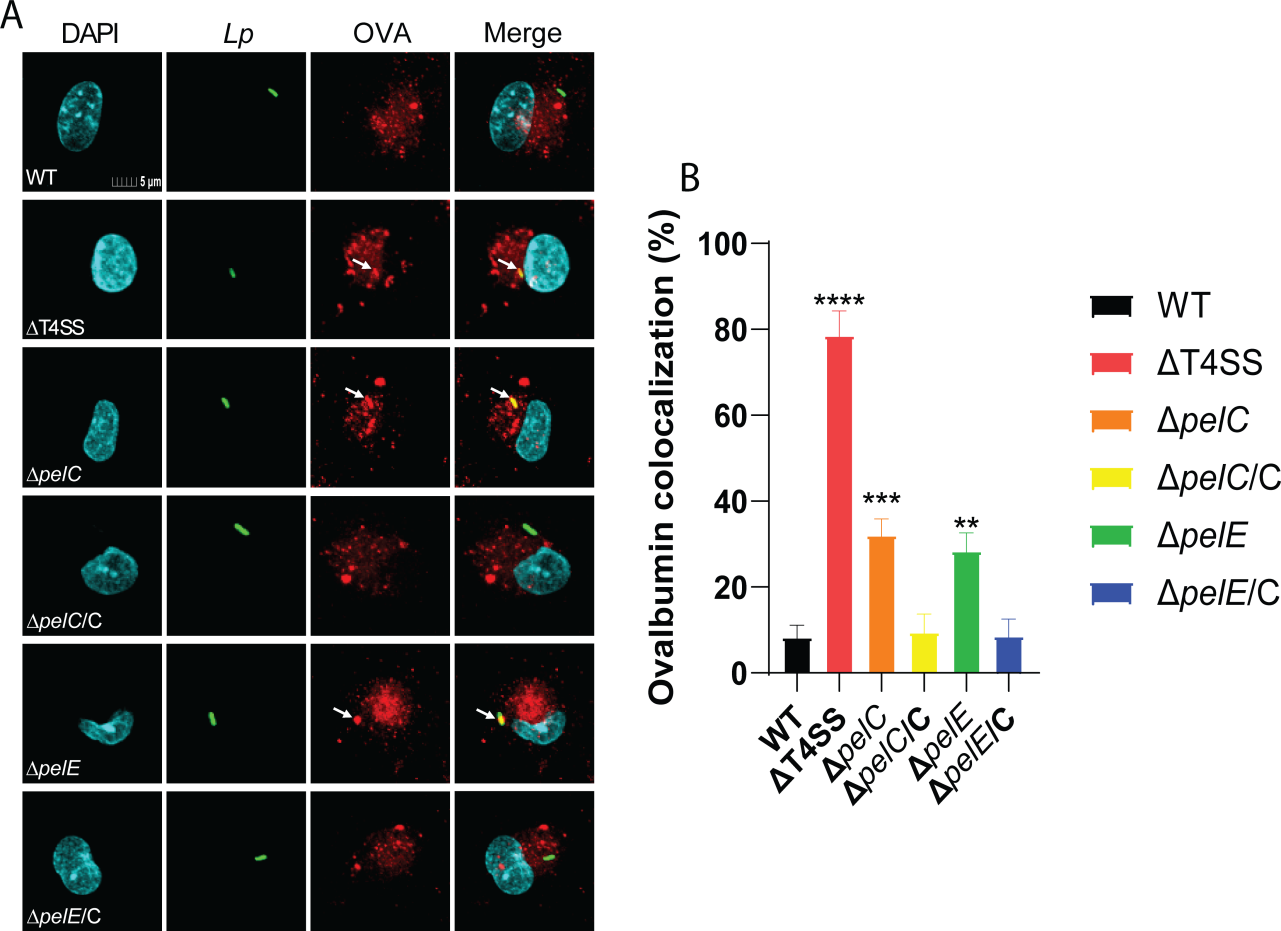
Role of PelC and PelE in evasion of lysosomal fusion. To determine whether PelC and PelE are required for lysosomal evasion of the LCV, hMDMs were preloaded with AlexaFluor 555-Ovalbumin and infected with WT, *ΔT4SS*, *ΔpelC*, *ΔpelE*, and the complemented *pelC*/C and *pelE*/E strains. (**A**) Representative images of colocalization of the LCVs with ovalbumin. Cells are labeled with DAPI (cyan), anti-*L*. *pneumophila* (green), and AlexaFluor 555-Ovalbumin (red). (**B**) Quantification of colocalization with the LCV. All analyses were performed on at least 100 infected cells from multiple coverslips. Data are shown as mean percent colocalization of ovalbumin with the LCVs ± SD (error bars) and are representative of three independent experiments performed in triplicates. Values that are significantly different by one-way ANOVA are indicated as follows: ** (*P* ≤ 0.01), *** (*P* ≤ 0.001), **** (*P* ≤ 0.0001).

In addition to the lysosomal enzyme, the lysosomes of hMDMs were preloaded with the lysosomal tracer Alexa Fluor 555-Ovalbumin for 90 min and chased for 30 min prior to infection to allow trafficking of ovalbumin to lysosomes (42). Cells were infected for 1 h and fixed, permeabilized, and analyzed by confocal microscopy. Consistent with the lysosomal enzyme colocalization, about 12% of WT LCVs colocalized with the lysosomal tracer, while 72% of *ΔT4SS* LCVs colocalized with the tracer (one-way ANOVA, *P* < 0.0001) (Fig. 7A and B). The data showed that 33% and 26% of LCVs of the *ΔpelC* and *ΔpelE* mutants, respectively, colocalized with the lysosomal tracer, which was significantly higher than LCVs of WT strain (one-way ANOVA, *P* < 0.0003, *P* < 0.007) (Fig. 7A and B). We conclude that the LCV-localized PelC and PelE effectors are involved in ER-mediated remodeling of the LCV and evasion of the endosomal-lysosomal pathway, which is consistent with the significant role of the two effectors in intracellular replication of *L. pneumophila* in hMDMs and *A. polyphaga*.

## DISCUSSION

Although *L. pneumophila* is known to have the largest repertoire of effector proteins among bacterial pathogens, the subcellular localization of most effectors during infection remains unknown (15). Bacterial effectors utilize multiple mechanisms to anchor themselves to cellular membranes to attain their biological functions within the host cell. For example, SidC in *L. pneumophila* targets specific phospholipids present on cellular membranes, such as phosphatidylinositol-4-phosphate, to achieve membrane anchoring (43). Other bacterial effectors, such as SseG in *Salmonella typhimurium*, possess a transmembrane domain that facilitates its localization to the Golgi apparatus (44). In addition, some effectors undergo post-translational lipid modifications, which enable their anchoring into host membranes by introducing hydrophobic moieties. LpdA in *L. pneumophila* is palmitoylated at a C-terminal cysteine residue to be localized to the LCV membrane (45). Some effectors of plant pathogens, such as AvrB and AvrRpm1 of *Pseudomonas syringae*, undergo myristoylation at the N-terminal GXXX- motif to localize to the plasma membrane of the host cells (46). The AnkB effector of *L. pneumophila* is prenylated at the C-terminal -CaaX motif to localize to the LCV membrane (24). Here, we show that, similar to AnkB, the seven Pels (PelA, PelC, PelD, PelE, PelH, PelI, and PelK) are localized to the cytosolic face of the LCV membrane in a Dot/Icm-dependent manner during infection of macrophages. Prenylation of the cysteine residue of the C-terminal -CaaX motif of the Pel effectors is required for their LCV localization and biological function in intracellular replication, similar to AnkB (24). Similarly, PelE/LegG1/MitF of strain JR32 localizes to the LCV during infection of *D. discoideum* (27). Different effectors of *Legionella* possess different Dot/Icm translocation signals located in various domains of the effectors. This includes a C-terminus signal, an “E-block”/glutamate-rich near the C-terminus, transmembrane domains (TMDs) signal, and IcmSW chaperone-dependent recognition signal (14, 47–52). Previous studies on a different prenylated effector, AnkB, have shown that mutations of the conserved cysteine residue in the C-terminal -CaaX motif do not affect translocation efficiency (25, 53). However, we cannot exclude the possibility that a substitution in the C-terminal region of some effectors might interfere with translocation efficiency (54). Importantly, these findings narrow the gap between exceptionally large Dot/Icm effector repertoire and the limited subset of effectors that are required for intracellular replication in primary human macrophages. While most individual effectors are dispensable, our data identify PelC and PelE as prenylation-dependent determinants of LCV biogenesis that directly contribute to bacterial fitness. This highlights host-mediated lipidation as a central organizing principle for localizing effectors at the LCV.

As an aquatic generalist pathogen, *L. pneumophila* has adapted to various protozoan hosts, which has enabled evolution of the bacterium to manipulate various conserved eukaryotic host cellular processes (2). The long-term coevolution of the bacterium with its protozoan hosts has led to the acquisition of protozoan host genes through interkingdom horizontal gene transfer (2, 55–58). Consequently, *L. pneumophila* encodes a diverse arsenal of effectors, essentially a toolbox, utilized in a host-specific manner (2). It is therefore not surprising that the role of certain effectors exhibits host tropism as their roles vary depending on the amoeba host. The MavF effector is required for intracellular replication of *L. pneumophila* in *A. castellanii* and *A. polyphaga*, but not in *V. vermiformis*, whereas RavO is essential for replication in *V. vermiformis* but dispensable in *A. castellanii* and *A. polyphaga* (59). Our data show that PelC and PelE are necessary for robust intracellular replication of *L. pneumophila* in hMDMs and *A. polyphaga*. However, PelC and PelE exhibit host tropism, as both are dispensable in *V. vermiformis*. A *ΔpelE/legG1/mitF* mutant of strain JR32 shows impaired intracellular replication of *L. pneumophila* during infection of RAW264.7 cells and *Acanthamoeba castellanii* (27). The role of PelE/LegG1/MitF in infection of human macrophages or other amoeba species is not known. Additionally, the role of some effectors in the intracellular infection is strain-specific. For example, deletion of the *ankB* gene impairs intracellular replication in strain AA100/130b and strain Paris, but AnkB is dispensable in strain Philadelphia (60–63). Importantly, while most *L. pneumophila* effectors are not required for intracellular replication in macrophages and protozoan hosts, our results demonstrate that PelC and PelE are required for intracellular replication of *L. pneumophila* in hMDMs and *A. polyphaga*. This host-specific requirement underscores how *L. pneumophila* leverages a flexible effector toolbox, whose components can be selectively advantageous in certain protozoan hosts but dispensable in others. Such context-dependent effector usage likely contributes to environmental persistence, amplification, and ultimately the enhanced capacity to infect humans.

A key aspect of LCV biogenesis is its ability to intercept ER secretory vesicles, transforming the LCV into an ER-derived vacuole (64). Several effectors contribute to this process, including RalF, SidM, LidA, SidD, MavE, and LepB (30, 35, 65–69). Our findings show that PelC and PelE play a role in ER-mediated remodeling of the LCV. However, the *ΔpelE/legG1/mitF* mutant did not show impaired recruitment of ER resident protein Calnexin on the LCV during infection of *D. discoideum* (27). Few effectors of *L. pneumophila*, such as VipD and MavE, have been shown to be involved in lysosomal evasion by the LCV (30, 36, 70–72). Our findings show that PelC and PelE contribute to evasion of the endosomal-lysosomal pathway by the LCV. Bioinformatic analyses revealed no detectable sequence or Alphafold2 structural similarities between PelC and PelE, indicating that these effectors are not functionally redundant. Further studies examining the host target and elucidating the mechanism of PelC will provide critical insight into its role in LCV remodeling.

We have shown that the seven Pel effectors (PelA, PelC, PelD, PelE, PelH, PelI, and PelK) localize to the cytosolic face of the LCV in a Dot/Icm-dependent manner through host-mediated prenylation of their highly conserved C-terminal -CaaX motif. The PelC and PelE are required for robust intracellular proliferation of *L. pneumophila* during infection of human macrophages, and both effectors exhibit host tropism as they are required in *A. polyphaga* but are dispensable in *V. vermiformis*. The PelC and PelE effectors contribute to ER-mediated remodeling of the LCV and evading lysosomal fusion. While most *L. pneumophila* effectors are dispensable for infection of macrophages, our findings identify two LCV-localized prenylated effectors, PelC and PelE, to be required for the infection of macrophages and some protozoan hosts, expanding the limited repertoire of effectors known to be required for the robust replication of *L. pneumophila* within human macrophages and protozoan hosts.

## MATERIALS AND METHODS

### Bacterial strains

*Legionella pneumophila* strain AA100/130b (ATCC BAA-74) and the T4SS-deficient mutant (*dotA*) were grown on BCYE agar plate as previously described (60). To generate isogenic *pel* mutants, 1.5 kb of DNA flanking both sides of the *pel* genes were amplified by PCR using the primers listed in Table 1 and cloned into a vector plasmid pBCSK+ (Stratagene). To delete the entire *pel* genes, inverse PCR was employed using primers listed in Table 1. The kanamycin resistance cassette was amplified from the pCR-Blunt II-TOPO (Thermo) using primers listed in Table 1, and the PCR product was subcloned into the inverse PCR product between the *pel*-flanking DNA regions using standard molecular biology techniques, resulting in the *pel* KO plasmids. The plasmids were transformed into *L. pneumophila* AA100 via natural transformation as described previously (73). To confirm the deletion of the *pel* genes, PCR was used using the primers listed in Table 1. To complement the *pelC* and *pelE* mutants, PCR was used to amplify the *pelC* and *pelE* genes using the primers listed in Table 1, and PCR products were subcloned into pBCSK+. The complement plasmids were transformed into the *pelC* and *pelE* mutants via electroporation as previously described (74). 4HA-Pels were generated as previously described (30). The complementation was possible in some of the experiments. This is due to the plasmids. The mCherry plasmid for image has the same origin of replication as the complementing plasmid, precluding their simultaneous use.

**TABLE 1:** Primers used in this study

Primer name	Nucleotide sequence
HA PelA F	ggtaccTTGAGTGAATATTTGGTTC
HA PelA R	ggatccCTACATGAGCACACAAACAGA
HA PelC F	ggtaccTTGATATTGGAATTTTCTGA
HA PelC R	ggatccTTAAATTATTGTGCAGCAAG
HA PelD F	ggatccGTGTTCAAAAAAAAGCATAT
HA PelD R	aagcttTCACAATAAAGAATAATTAT
HA PelE F	ggtaccTTGAGTCTTGCATCCTACAA
HA PelE R	ggatccTCATAGCAAATTACATGGCG
HA PelH F	ggtaccGTGCTAATGGAATTCGAAGA
HA PelH R	ggatccTTACATTATTGTACAACGG
HA PelI F	ggtaccATGAAATTTAAGATTGCAAC
HA PelI R	ggatccTTACCATATGATGCAACGAT
Ha PelK F	ggtaccATGTATACAAAAAATTCTCT
HA PelK R	ggatccTCAAGAAATCACGCAAGCAT
PelA CA F	ggtaccTTGAGTGAATATTTGGTTCT
PelA CA R	ggatccCTACATGAGCACAGCAACAG
PelC CA F	/5Phos/GCTACAATAATTTAAGGATC
PelC CA R	/5Phos/GCAAGGCGGCTCTCCTTCCT
PelD CA F	/5Phos/GCTTCTTTATTGTGAAAGCT
PelD CA R	/5Phos/ATTATTTGAGCTGATAATAC
PelE CA F	ggtaccTTGAGTCTTGCATCCTACAA
PelE CA R	ggatccTCATAGCAAATTAGCTGGCG
PelH CA F	ggtaccGTGCTAATGGAATTCGAAGA
PelH CA R	ggatccTTACATTATTGTAGCACGGT
PelI CA F	/5Phos/GCTATCATATGGTAAGGATC
PelI CA R	/5Phos/ACGATTCTTACTTATCATAG
PelK CA F	ggtaccATGTATACAAAAAATTCTCT
PelK CA R	ggatccTCAAGAAATCACGGCAGCAT

### Intracellular replication in hMDMs and amoeba

Intracellular replication studies of *L. pneumophila* strains in hMDMs, *A. polyphaga*, *and V. vermiformis* were performed as previously described (75). All methods were carried out and approved in accordance with the University of Louisville Institutional Review Board guidelines, and blood donors gave informed consent as required by the University of Louisville Institutional Review Board (IRB # 04.0358). Briefly, hMDMs were isolated from healthy donors and cultured in RPMI 1640 supplemented with 10% fetal bovine serum. *A. polyphaga* was cultured in PYG medium, and *V. vermiformis* was grown in ATCC 1034 medium as previously described.

The WT strain, *ΔT4SS* and *Δpel* isogenic mutants, and *ΔpelC/*C and *ΔpelE/*E complemented strains were grown to post-exponential phase in BYE broth at 37°C with shaking. These strains were used to infect hMDMs, *A. polyphaga*, and *V. vermiformis*. A total of 1 × 10^5^ host cells were plated in 96-well plates and infected with *L. pneumophila* strains at a multiplicity of infection (MOI) of 10. The plates were centrifuged at 200 × *g* (hMDMs) or 600 × *g* (amoeba) for 5 min to synchronize infection and incubated at 37°C. After 1 h post-infection, cells were treated with gentamicin (50 µg/mL) for 1 h to kill extracellular bacteria. Host cells were lysed with sterile water (hMDMs) or 0.04% Triton X-100 (amoeba) at 2, 8, 24, and 48 h post-infection. Bacterial CFUs were determined by plating serial dilutions onto BCYE agar plates. The infection experiments using *ΔpelA*, *ΔpelC*, *ΔpelD*, *ΔpelE*, *ΔpelH*, and the *pelC-* and *pelE*-complemented strains were done at the same time, and experiments using *ΔpelI* and *ΔpelK* were carried out separately because generating these mutants took longer.

### Confocal microscopy

Processing of infected cells for confocal microscopy was performed as previously described (30). 2 × 10^5^ hMDMs were prepared using the same protocol for intracellular replication on coverslips in 24-well plates. The monolayers were infected with *L. pneumophila* at an MOI of 10. The plates were centrifuged at 200 × *g* for 5 min to synchronize infection and incubated at 37°C. After 1 h post-infection, cells were treated with gentamicin (50 µg/mL) for 1 h to kill extracellular bacteria.

To determine intracellular localization of 4HA-tagged Pels, cells were infected with post-exponential-phase WT or *ΔT4SS* that had 4HA-tagged Pel constructs. At 8 h post-infection, cells were fixed in 4% PFA for 30 min and permeabilized with 0.1% Triton X-100 in PBS for 10 min or fixed and permeabilized with methanol for 5 min. Cells were labeled with goat anti-*L*. *pneumophila* anti-serum (1:1,000) (76) and detected by Alexa Fluor 488-conjugated donkey anti-goat IgG (1:2,000, Invitrogen) and mouse anti-HA (1:500, Invitrogen) and detected by Alexa Fluor 555-conjugated donkey anti-mouse IgG (1:2,000, Invitrogen). To determine if prenylation machinery is responsible for intracellular localization of 4HA-tagged Pels, the cysteine residue of the -CaaX motif was substituted to alanine. Cells were infected and processed the same way.

To determine if PelC and PelE are involved in intracellular trafficking of the LCV, hMDMs were infected with post-exponential-phase WT, *ΔT4SS*, *ΔpelC*, *ΔpelE*, *ΔpelC/*C, or *ΔpelE/*E at a MOI of 10. At 2 h post-infection, cells were fixed in −20°C methanol for 5 min. Cells were labeled with goat anti-*L*. *pneumophila* anti-serum (1:1,000) and detected by Alexa Fluor 488-conjugated donkey anti-goat IgG (1:2,000, Invitrogen); rabbit anti-Cathepsin D (1:250, Invitrogen) and detected by Alexa Fluor 555-conjugated donkey anti-rabbit IgG (1:2,000, Invitrogen); and rabbit anti-Calnexin (1:250, Proteintech) and detected by Alexa Fluor 555-conjugated donkey anti-rabbit IgG (1:2,000, Invitrogen). Alexa Fluor 555-conjugated ovalbumin (100 µg/mL, Invitrogen) was preloaded to hMDMs for 1.5 h and chased for 30 min after washing with PBS before infection. LysoTracker Green DND-26 (50 nM, Invitrogen) was added to hMDMs for 30 min and washed with PBS. Cells were infected with mCherry-expressing bacteria for 30 min at a MOI of 100, and live-cell images were taken.

All confocal microscopy images were taken as Z-stacks of 10–15 slices in 0.5 µm slices.

### Western blot

*L. pneumophila* strains that produce the HA-Pels fusions were grown in BYE broth at 37°C. Once the cultures reached an optical density (OD_550_) of ~1.0, they were induced with 1 mM IPTG and grown to post-exponential phase (OD ~2.0). Samples were prepared by resuspending 10^7^ bacterial cells in 1× Laemmli sample buffer (Bio-Rad) and heated at 95°C for 10 min. Samples were separated on 8–16% SDS Mini-PROTEAN TGX Precast Gels (Bio-Rad). Proteins were transferred to a polyvinylidene difluoride (PVDF) membrane using Trans-Blot Turbo Transfer System and probed with mouse anti-HA (1:5,000, Invitrogen). Fusion proteins were detected via anti-mouse-HRP conjugates and developed by chemiluminescence.

### Statistical analysis

Data are presented as mean and SD of three replicates. Statistical significance was determined using Student’s *t*-test and ANOVA. Statistical analysis was performed in GraphPad Prism 10.

## Data Availability

Original contributions presented in the study are included in the article and its Supplemental material. Further inquiries can be directed to the corresponding author.

## SUPPLEMENTAL MATERIAL

Supplemental figures iai.00605-25-s0001.pdf Fig. S1 to S6.

ASM does not own the copyrights to Supplemental Material that may be linked to, or accessed through, an article. The authors have granted ASM a non-exclusive, world-wide license to publish the Supplemental Material files. Please contact the corresponding author directly for reuse.
