# Supplementary material for: Localization of the PelC and PelE effectors to the Legionella-containing vacuole through host-mediated prenylation and their role in intracellular proliferation
Source: Infect Immun. 2026 Mar 24;94(4):e00605-25. doi: 10.1128/iai.00605-25 (PMC13081727; doi:10.1128/iai.00605-25)
Supplement: Supplemental figures — Fig. S1 to S6. [file iai.00605-25-s0001.pdf]

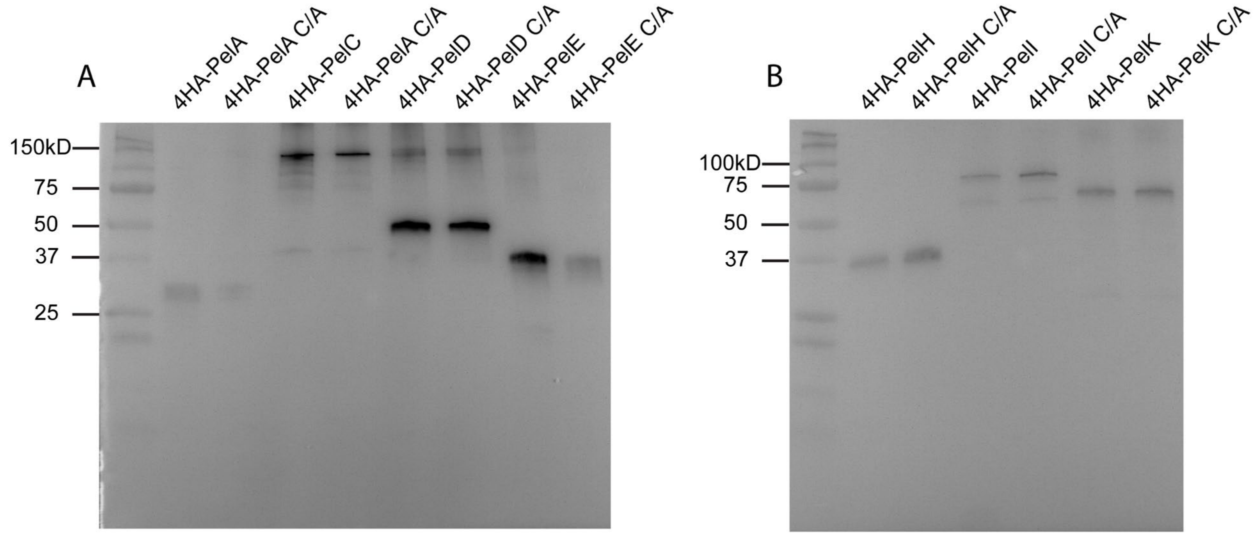

Supplementary Figure 1. Production of cysteine substituted 4HA-Pels. To determine the production of cysteine substituted 4HA-Pels, cysteine residue of the C-terminal -CaaX motif were substituted to alanine. 4HA-Pel C/A fusion plasmids were constructed with an IPTG-inducible promoter and transformed into WT *L. pneumophila*. **(A)** Western blot of total cell lysates from *L. pneumophila* strains harboring 4HA-PelA, 4HA-PelC, 4HA-PelD, and 4HA-PelE and the cysteine substituted plasmids. **(B)** Western blot of total cell lysates from *L. pneumophila* strains harboring 4HA-PelH, 4HA-PelI, and 4HA-PelK and the cysteine substituted plasmids.

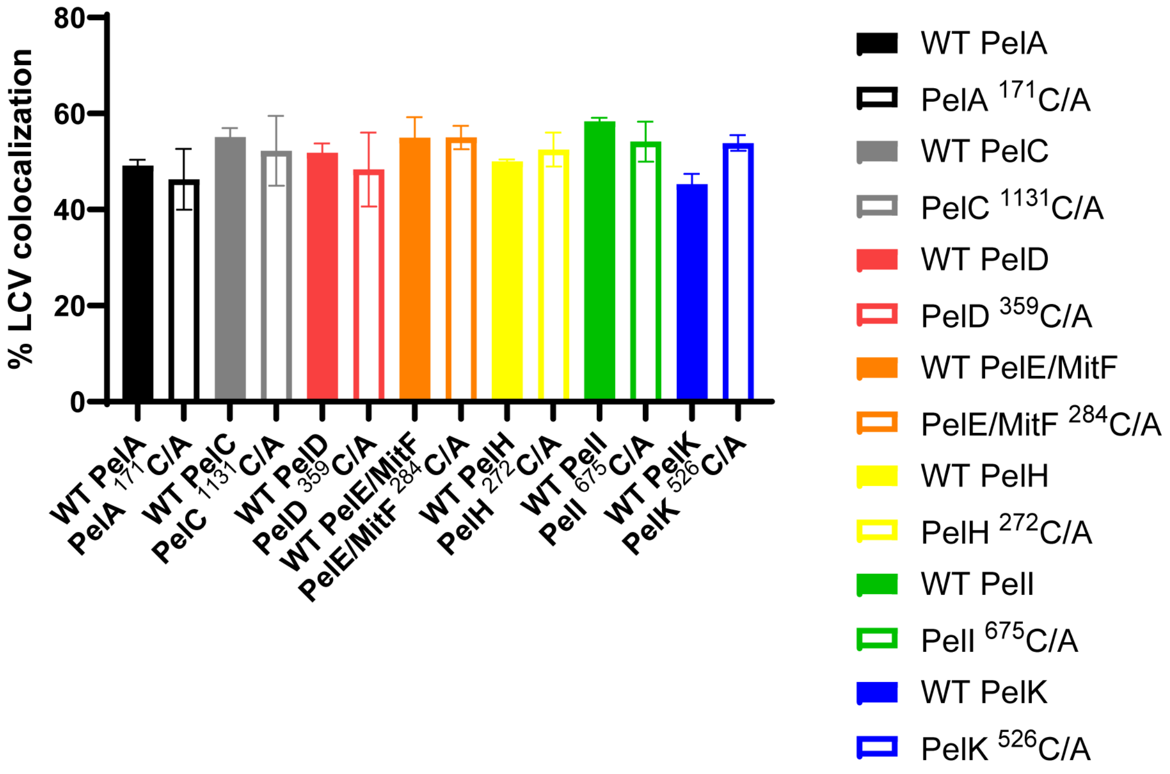

Supplementary Figure 2. Localization of Pels during hMDM infection with methanol treatment. To confirm triton treatment does not permeabilize all the membrane, the subcellular localization of the Pels during the infection of hMDMs was analyzed after methanol treatment. 4HA-tagged WT and C/A Pels constructs were transformed into WT *L. pneumophila*. Images are representative confocal microscopy images of 4HA-Pels (red) colocalizing with the bacteria (green). All analyses were performed on at least 100 infected cells from multiple coverslips. Data are shown as mean percent colocalization of HA-tagged fusion proteins with the LCVs ± standard deviations (SD) and are representative of three independent experiments done in triplicates.

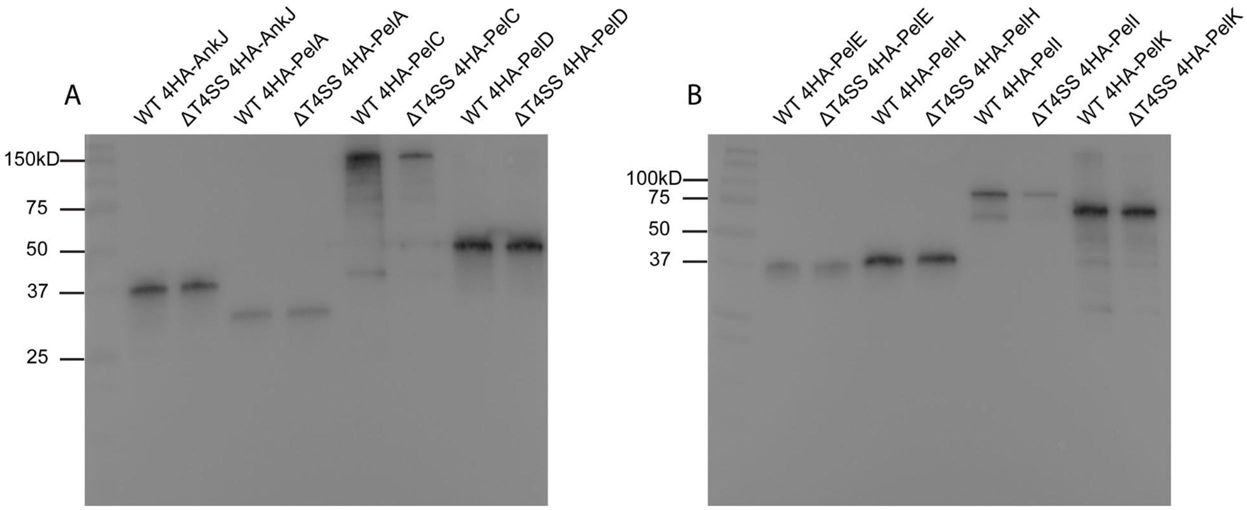

Supplementary Figure 3. Production of 4HA-Pels. To determine the production of 4HA-Pels, 4HA-Pels fusion plasmids were constructed with an IPTG-inducible promoter and transformed into WT and *ΔT4SS L. pneumophila*. **(A)** Western blot of total cell lysates from *L. pneumophila* strains harboring 4HA-AnkJ, 4HA-PelA, 4HA-PelC, and 4HA-PelD. **(B)** Western blot of total cell lysates from *L. pneumophila* strains harboring 4HA-PelE, 4HA-PelH, 4HA-PelI, and 4HA-PelK.

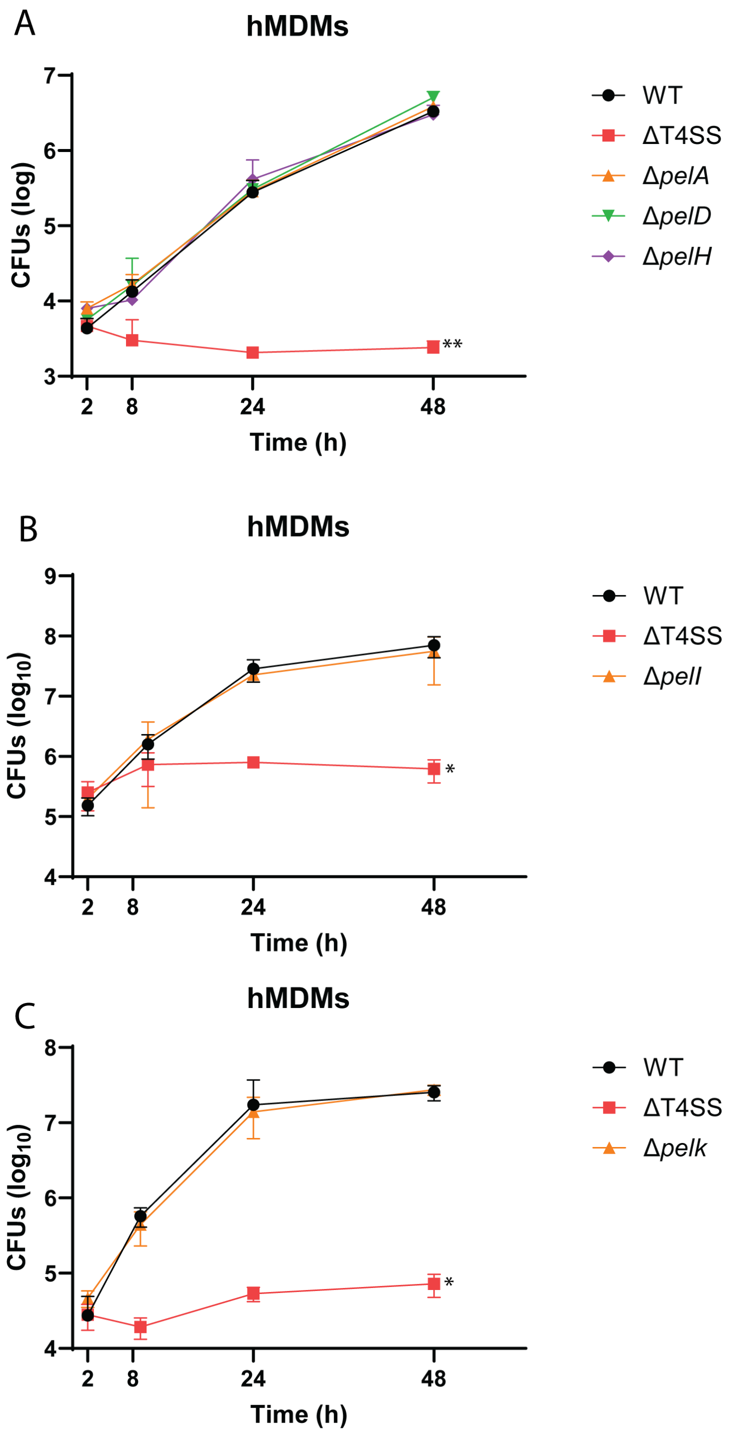

Supplementary Figure 4. All the Pels except PelC and PelE are dispensable for intracellular replication of *L. pneumophila* in hMDMs. To determine intracellular replication of the WT, *ΔT4SS*, and the pel mutant strains, hMDMs were infected. The number of CFU was determined at 2, 8, 24, and 48 h postinfection. Data points represent mean CFU ± standard deviations (SD) (error bars) and are representative of at least three independent experiments done in triplicates. **(A)** CFUs of *ΔpelA, ΔpelD, ΔpelH*. **(B)** CFUs of *ΔpelI*. **(C)** CFUs of *ΔpelK*. Values that are significantly different by Student *t* test are indicated as follows: * ($P \leq 0.05$), ** ($P \leq 0.01$)

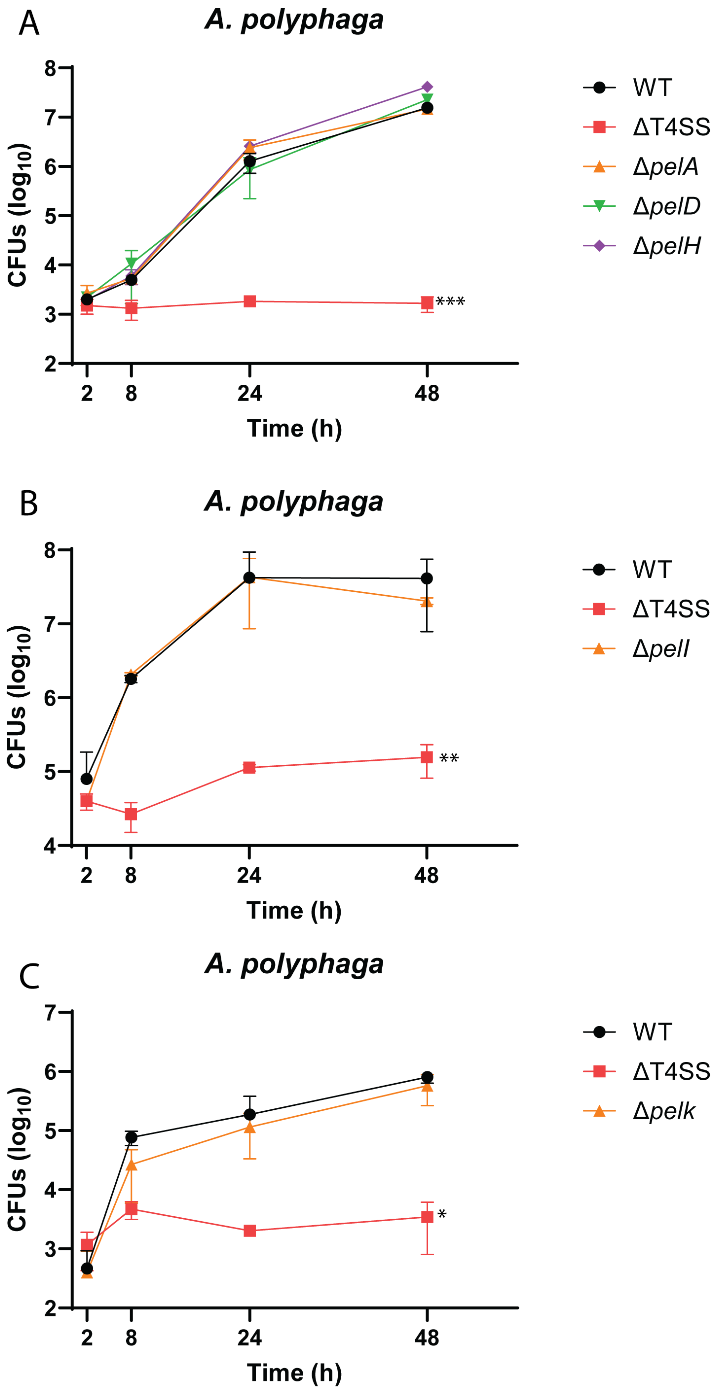

Supplementary Figure 5. All the Pels except PelC and PelE are dispensable for intracellular replication of *L. pneumophila* in *A. polyphaga*. To determine intracellular replication of the WT, *ΔT4SS*, and the pel mutant strains, *A. polyphaga* were infected. The number of CFU was determined at 2, 8, 24, and 48 h postinfection. Data points represent mean CFU ± standard deviations (SD) (error bars) and are representative of at least three independent experiments done in triplicates. **(A)** CFUs of *ΔpelA, ΔpelD, ΔpelH*. **(B)** CFUs of *ΔpelI.* **(C)** CFUs of *ΔpelK*. Values that are significantly different by Student *t* test are indicated as follows: * ($P \leq 0.05$), ** ($P \leq 0.01$), *** ($P \leq 0.001$).

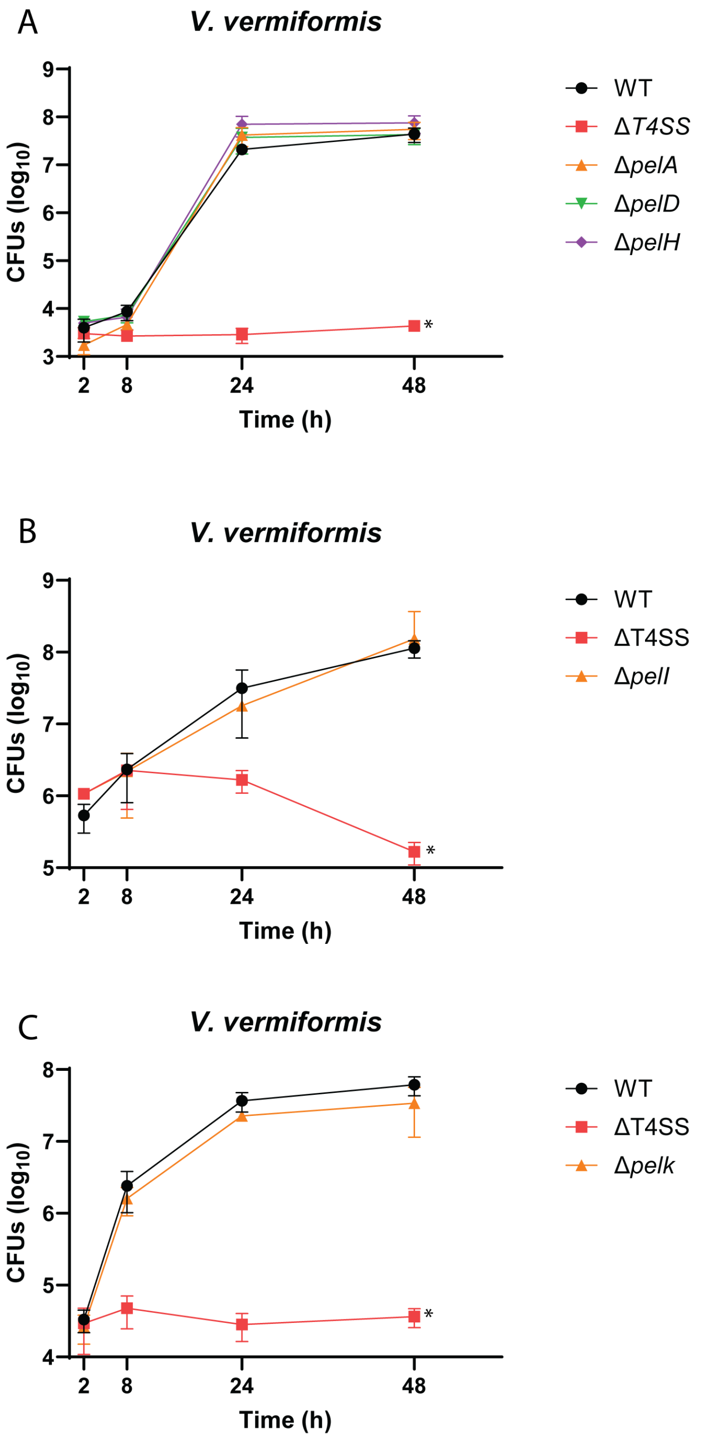

Supplementary Figure 6. All the Pels are dispensable for intracellular replication of *L. pneumophila* in *V. vermiformis*. To determine intracellular replication of the WT, *ΔT4SS*, and the pel mutant strains, *V. vermifornis* were infected. The number of CFU was determined at 2, 8, 24, and 48 h postinfection. Data points represent mean CFU ± standard deviations (SD) (error bars) and are representative of at least three independent experiments done in triplicates. **(A)** CFUs of *ΔpelA, ΔpelD, ΔpelH*. **(B)** CFUs of *ΔpelI*. **(C)** CFUs of *ΔpelK*. Values that are significantly different by Student *t* test are indicated as follows: * ($P \leq 0.05$)
